# Lung-Protective Ventilation Attenuates Mechanical Injury While Hypercapnia Attenuates Biological Injury in a Rat Model of Ventilator-Associated Lung Injury

**DOI:** 10.3389/fphys.2022.814968

**Published:** 2022-04-21

**Authors:** Nada Ismaiel, Sara Whynot, Laurette Geldenhuys, Zhaolin Xu, Arthur S. Slutsky, Valerie Chappe, Dietrich Henzler

**Affiliations:** ^1^ Faculty of Medicine, Dalhousie University, Halifax, NS, Canada; ^2^ Department of Anesthesia, Faculty of Medicine, University of Toronto, Toronto, ON, Canada; ^3^ Department of Anesthesia, Faculty of Medicine, Dalhousie University, Halifax, NS, Canada; ^4^ Department of Pathology, Faculty of Medicine, Dalhousie University, Halifax, NS, Canada; ^5^ Faculty of Medicine, University of Toronto, Toronto, ON, Canada; ^6^ Department of Physiology and Biophysics, Faculty of Medicine, Dalhousie University, Halifax, NS, Canada; ^7^ Department of Anesthesiology, Medical Faculty, Ruhr University Bochum, Bochum, Germany

**Keywords:** lung-protective mechanical ventilation, hypercapnia, ventilator associated lung injury, acute lung injury, mechanical ventilalion

## Abstract

**Background and Objective:** Lung-protective mechanical ventilation is known to attenuate ventilator-associated lung injury (VALI), but often at the expense of hypoventilation and hypercapnia. It remains unclear whether the main mechanism by which VALI is attenuated is a product of limiting mechanical forces to the lung during ventilation, or a direct biological effect of hypercapnia.

**Methods:** Acute lung injury (ALI) was induced in 60 anesthetized rats by the instillation of 1.25 M HCl into the lungs via tracheostomy. Ten rats each were randomly assigned to one of six experimental groups and ventilated for 4 h with: 1) **Conventional HighV**_**E**_ **Normocapnia** (high V_T_, high minute ventilation, normocapnia), 2) **Conventional Normocapnia** (high V_T_, normocapnia), 3) **Protective Normocapnia** (V_T_ 8 ml/kg, high RR), 4) **Conventional iCO**_**2**_ **Hypercapnia** (high V_T_, low RR, inhaled CO_2_), 5) **Protective iCO**_**2**_ **Hypercapnia** (V_T_ 8 ml/kg, high RR, added CO_2_), 6) **Protective endogenous Hypercapnia** (V_T_ 8 ml/kg, low RR). Blood gasses, broncho-alveolar lavage fluid (BALF), and tissue specimens were collected and analyzed for histologic and biologic lung injury assessment.

**Results:** Mild ALI was achieved in all groups characterized by a decreased mean PaO_2_/FiO_2_ ratio from 428 to 242 mmHg (*p* < 0.05), and an increased mean elastance from 2.46 to 4.32 cmH_2_O/L (*p* < 0.0001). There were no differences in gas exchange among groups. Wet-to-dry ratios and formation of hyaline membranes were significantly lower in low V_T_ groups compared to conventional tidal volumes. Hypercapnia reduced diffuse alveolar damage and IL-6 levels in the BALF, which was also true when CO_2_ was added to conventional V_T_. In low V_T_ groups, hypercapnia did not induce any further protective effect except increasing pulmonary IL-10 in the BALF. No differences in lung injury were observed when hypercapnia was induced by adding CO_2_ or decreasing minute ventilation, although permissive hypercapnia decreased the pH significantly and decreased liver histologic injury.

**Conclusion:** Our findings suggest that low tidal volume ventilation likely attenuates VALI by limiting mechanical damage to the lung, while hypercapnia attenuates VALI by limiting pro-inflammatory and biochemical mechanisms of injury. When combined, both lung-protective ventilation and hypercapnia have the potential to exert an synergistic effect for the prevention of VALI.

## Introduction

The role of lung-protective ventilation using low inspiratory pressures and low tidal volumes for attenuating ventilator-associated lung injury (VALI) is well established (Amato et al., 1998; Acute Respiratory Distress Syndrome et al., 2000; Slutsky and Ranieri, 2013). Protective ventilation is believed to attenuate VALI by limiting barotrauma and volutrauma, thereby reducing the stretch and strain to lung tissue during the inflation phase of the respiratory cycle. Barotrauma and volutrauma have been specifically attributed to mechanical power, which is directly proportional to the respiratory rate and transpulmonary driving pressures, and inversely proportional to lung elastance (Amato et al., 2015). Santos et al. (2018) recently demonstrated that increased ventilatory power, which is primarily a product of increased transpulmonary driving pressure, worsened VALI as expected. The transpulmonary pressure is calculated from the difference between airway and intrapleural pressure, measured clinically in the mid-esophagus. The transpulmonary driving pressure is derived from inspiratory and expiratory transpulmonary pressures which determine the achieved tidal volume. If no esophageal pressure is available, the airway driving pressure may be used as a surrogate and has been shown to correlate with outcome (Amato et al., 2015).

Two mechanisms of injury have been the focus of research in the field of VALI: 1) The direct structural damage caused by overdistension of lung units leading to volutrauma, barotrauma, and 2) biotrauma (i.e., the activation of biological pathways via mechanotransduction). Cyclic opening and closure of distal airways/alveoli during the ventilatory cycle, termed atelectrauma, may contribute to all of the above by increasing stress and strain. When ventilatory power and driving pressures are reduced using lung protective ventilation (Rocco et al., 2012; Santos et al., 2018) there may be a reduction in structural injury (pulmonary edema and histologic lung injury), a reduction in biotrauma (pro-inflammatory mediators) and improved clinical outcomes (Santos et al., 2018).

One of the consequences of limiting driving pressure, tidal volume ventilation and respiratory rate is the rise in arterial PaCO_2_, a concept known as permissive hypercapnia (Hickling et al., 1994; Contreras et al., 2015; Costa et al., 2021). Although the differential mechanisms of biological vs mechanical effects of permissive hypercapnia have not been systematically investigated, several clinical studies have investigated lung protective ventilation with permissive hypercapnia as a tolerated side effect (Bidani et al., 1994; Hickling et al., 1994; Acute Respiratory Distress Syndrome et al., 2000). Hypercapnia has been associated with a reduction in the effects of excessive lung stretch by an intracellular mechanism that remains elusive (Ismaiel and Henzler, 2011). In view of the clinical benefits, lung protective ventilation allowing permissive hypercapnia has been adopted in current treatment guidelines for acute respiratory distress syndrome (ARDS), as well as neonatal respiratory failure, acute status asthmaticus (Darioli and Perret, 1984), and respiratory failure secondary to chronic obstructive pulmonary disease (Contreras et al., 2015).

The hypothesis that hypercapnia may no longer be viewed as a side effect but rather as a therapeutic concept (Kavanagh and Laffey, 2006) by adding a small fraction of inspired carbon dioxide (CO_2_) to the gas mixture during ventilation was experimentally introduced in recent years (Ismaiel and Henzler, 2011). This therapeutic hypercapnia has been shown to attenuate pulmonary inflammation and free radical production, in addition to preserving pulmonary mechanics in an ischemia reperfusion-induced lung injury model in rabbits (Laffey et al., 2000). The resulting hypercapnic acidosis is characterized by a decrease in intracellular pH due to the accumulating CO_2_ and has been associated with a reduction in pulmonary inflammation, oxidative stress (Broccard et al., 2001) and cell death (Laffey et al., 2000; Chonghaile et al., 2008). Specifically, there is evidence to suggest that hypercapnic acidosis reduces Xanthine Oxidase activity, thereby reducing the production of free radicals and reactive oxygen species (Shibata et al., 1998; Ismaiel and Henzler, 2011).

Further, antimicrobial and anti-inflammatory properties have been attributed to hypercapnia as evidenced by reduced inflammatory cell infiltrates and bacterial cell counts in the injured lungs (Chonghaile et al., 2008). The acidotic state resulting from hypercapnia is associated with a reduction in architectural damage and histologic injury (Chonghaile et al., 2008), decreased pulmonary edema (Sinclair et al., 2002) and reduced production of key pro-inflammatory mediators, including IL-1β, TNF-α, IL-6, MCP-1, MMP-9, and KC (Laffey et al., 2000; Peltekova et al., 2010). Potential harmful effects of hypercapnia and hypercapnic acidosis have been described to occur in impaired cell membrane and wound healing, depressed cardiac function and uncontrolled increase in intracerebral pressure in case of brain injury (Ismaiel and Henzler, 2011). Despite the potentially therapeutic and protective effects of experimental hypercapnic acidosis noted previously, the true effect of acidosis has yet to be robustly studied in the clinical setting. It is well known that the acidotic state secondary to respiratory failure seen in ALI and ARDS is associated with deleterious effects and poor clinical outcomes (Tiruvoipati et al., 2017). Since the acidotic state in ALI and ARDS is often a mixture of respiratory and metabolic acidosis induced by multi-organ dysfunction, trying to compensate by hyperventilation increases VALI, while buffering the acidotic state with sodium bicarbonate remains controversial and potentially harmful (Chand et al., 2021). However, the true effect of hypercapnic acidosis either in the context of permissive hypercapnia or by adding a small fraction of inspired carbon dioxide (CO_2_) to the gas mixture during ventilation remains elusive.

To date it’s unclear whether the attenuation of VALI with lung protective ventilation and associated hypercapnia can be attributed mainly to reduction of the mechanical forces to the lung, to a direct effect of hypercapnia and acidosis, or a combination thereof. The aim of our study was thus to address five key questions:

1. Does lung-protective ventilation with low tidal volumes and limited driving pressures cause less VALI than conventional non-protective ventilation (i.e., higher tidal volumes) during normocapnia?
2. Does lung protective ventilation with permissive hypercapnia cause less VALI than normocapnic lung-protective ventilation?
3. Does exogenous CO_2_ attenuate VALI during ventilation with high tidal volumes?
4. Does exogenous CO_2_ attenuate VALI during lung protective ventilation with low tidal volumes?
5. Is there a different effect if hypercapnia is produced by exogenous delivery of carbon dioxide instead of permissive hypercapnia with endogenous rise in CO_2_?

## Materials and Methods

### Experimental Procedures

All experimental procedures were conducted with ethics approval from the Dalhousie University Committee on Laboratory Animals, and the care and handling of the animals was in accordance with the National Institutes of Health Guidelines for ethical animal treatment. Sixty male Sprague-Dawley rats (15–18 weeks old, weight 400–490 g) were used. Details of experimental procedures are outlined in the Supplementary Material.

In brief, anesthetized animals received a continuous IV infusion of 20 mcg/ml remifentanil and 25 mcg/ml pancuronium at 5 ml/h during controlled ventilation as described elsewhere (Ismaiel et al., 2012). Animals were tracheotomized with a 14G cannula and mechanically ventilated (EVITA4, Draeger Medical Canada Inc., Richmond, ON, Canada). The carotid artery and internal jugular vein were cannulated with 20G catheters for blood pressure monitoring and blood gas analysis (ABL510 and OSM3, Radiometer Copenhagen, Denmark). The femoral artery was cannulated with a thermocouple probe (ADInstruments Inc., Colorado Springs, CO, United States) for cardiac output measurements and cardiac index calculations. A pneumotachometer (Hans Rudolph Inc., Shawnee, KS, United States) was used to measure flow-related respiratory variables. A complete set of measurements was taken at baseline, and after 1 and 4 h of ventilation. The parameters measured at each time point included hemodynamics (MAP, CI, HR), respiratory variables (V_T_, RR, V_E_) and arterial blood gasses with 300 µl of blood per sample (PaO_2_, PaCO_2_, pH) with a FiO_2_ of 1.0 (Ismaiel et al., 2012).

We used a model of mild ARDS that had been previously described in rats and rabbits (Imai et al., 2003; Henzler et al., 2011) (see Supplementary Material). After baseline measurements, acute lung injury (ALI) was induced by endotracheal instillation of 2.5 ml/kg of unbuffered hydrochloric acid (HCl, pH 1.25), and allowed to develop over 1 hour of controlled ventilation with tidal volume (V_T_) 8 ml/kg, positive end expiratory pressure (PEEP) 5 cmH_2_O and a partial pressure of carbon dioxide (PaCO_2_) 40–55 mmHg, which was set by varying the respiratory rate. Establishment of ALI was defined by a PaO_2_:FiO_2_ (P/F) ratio ≤300 mmHg and a significant increase in respiratory system elastance.

Sixty rats were randomly assigned to receive one of six ventilation regimens (*n* = 10/group), each lasting 3 h. Three groups were ventilated to achieve relatively normocapnic conditions (PaCO_2_ = 45–55 mmHg) and three groups to achieve hypercapnic conditions (PaCO_2_ = 60–70 mmHg). All animals received a PEEP of 5 cmH_2_O while being ventilated (Table 1, 3).

**TABLE 1:** Summary of target ventilation settings, including tidal volume (V_T_, ml/kg), minute ventilation (V_E_), partial arterial pressure of carbon dioxide (PaCO_2_, mmHg), and the addition of inspired CO_2_ gas or dead space.

Group	V_T_ target (ml/kg)	V_E_	PaCO_2_ target (mmHg)	Additional CO_2_ or dead space
Conventional HighV_E_ Normocapnia	High (12)	⇧	40–55	1 ml added dead space
Conventional Normocapnia	High (12)	⇔	40–55	
Protective Normocapnia	Low (8)	⇔	40–55	
Conventional iCO_2_ Hypercapnia	High (12)	⇔	60–70	Inspired CO_2_ (1.6%)
Protective iCO_2_ Hypercapnia	Low (8)	⇔	60–70	Inspired CO_2_ (1.6%)
Protective Endogenous Hypercapnia	Low (8)	⇩	60–70	

#### Conventional HighV_E_ Normocapnia

Ventilation with high tidal volume (V_T_ 12 ml/kg) and increased minute ventilation (V_E_). 1 ml of dead space was added to the respiratory circuit to prevent hypocapnia and respiratory rate (RR) adjusted to achieve normocapnia (RR was 72 ± 16 breaths per minute).

#### Conventional Normocapnia

Ventilation with high V_T_ (12 ml/kg) and RR adjusted to maintain normocapnia (RR was 42 ± 10 breaths per minute).

#### Protective Normocapnia

Ventilation with low V_T_ (8 ml/kg) and RR adjusted to maintain normocapnia (RR was 92 ± 14 breaths per minute).

#### Conventional iCO_2_ Hypercapnia

Maintained V_E_ with high V_T_ (12 ml/kg) and inhaled CO_2_ (FiCO_2_ 1.6%) targeting hypercapnia (RR was 42 ± 11 breaths per minute).

#### Protective iCO_2_ Hypercapnia

Maintained V_E_ with low V_T_ (8 ml/kg) and inhaled CO_2_ (FiCO_2_ 1.6%) targeting hypercapnia (RR was 69 ± 19 breaths per minute).

#### Protective Endogenous Hypercapnia

Reduced V_E_ with low V_T_ (8 ml/kg) and low RR, with endogenous rise in PaCO_2_ by hypoventilation (RR was 52 ± 26 breaths per minute).

After final measurements and sample collection animals were killed with a 1 ml intravenous bolus of potassium chloride (150 mg/ml).

### Tissue and Fluid Analyses

Lung tissue samples were evaluated using the Diffuse Alveolar Damage (DAD) scoring criteria (Castro, 2006; Henzler et al., 2011). The liver and kidney tissue samples were graded for tissue damage scores on a scale from 0–2 and 0–4, respectively (See Supplementary Material). The wet-to-dry lung ratio was calculated from the weight of the wet right middle lobe after excision, and the dry weight after a 48-h incubation at 40°C. Lung homogenates were prepared, and the Bradford assay was used to prepare the samples for western blot analysis to quantify Caspase-3 protein expression (Kruger and Walker, 2002). Caspase-3 protein expression in the lung homogenates was expressed as the ratio of active (17 kD band) to inactive (35 kD band) caspase-3 (See Supplementary Material).

The BALF and arterial plasma samples were analyzed using a multiplex immunoassay (BIO-RAD; Hercules, California, United States). Cytokines and chemokines (interleukin (IL)-1β, ICAM-1, IL-6, TNF-α, GM-CSF, IL-10, RANTES, KC, MCP-1, and MIP-1α) were analyzed using the Luminex Technology Analyzer 100 and BioPlex Manager software from BIO-RAD (Mississauga, ON, Canada).

Data are expressed as means ± SD or SEM. All statistical analyses were conducted using GraphPad Prism 5.0 software (La Jolla, CA, United States). Normality of data was tested with K-S test. Differences between groups and changes over time of physiologic variables were tested by two-way ANOVA with a mixed-effects model, biologic variables by one-way ANOVA or Kruskal–Wallis test, whichever was appropriate. Adjustments for multiple comparisons was done by the Bonferroni method for adjusted *p* values. The level of significance was set at *p* < 0.05.

## Results

### Physiologic Measurements

**FIGURE 1:**
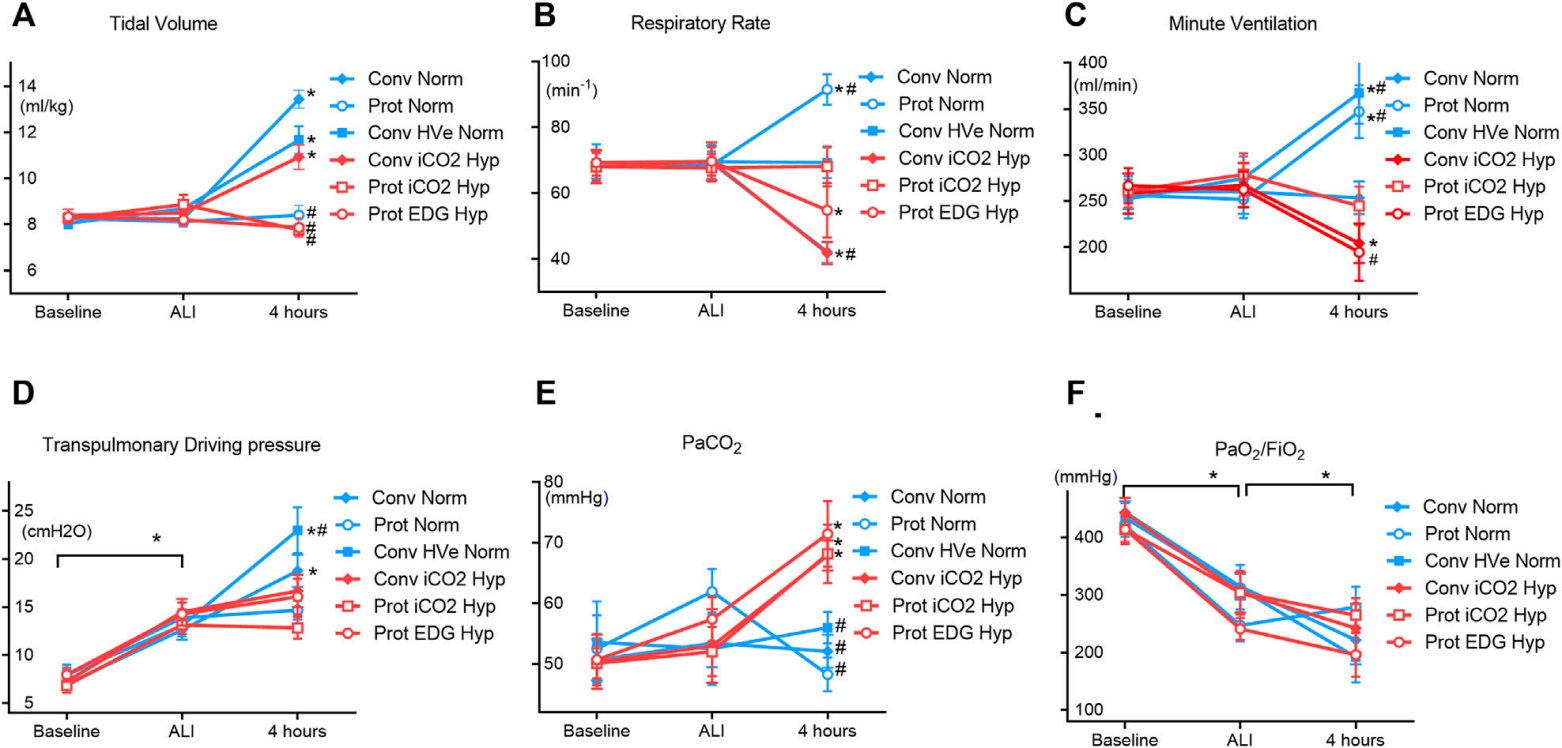
Physiologic dataat Baseline, ALI, and 4 h of ventilation (Mean ± SEM). Closed symbols: high tidal volume; open symbols: low tidal volumes; blue color: normocapnia; red color: hypercapnia. * denotes significant differences between groups.

Compared to baseline, 1 hour after induction of ALI the P/F ratio had decreased (428 ± 72 vs 287 ± 100 mmHg, *p* < 0.0001) and elastance (2.5 ± 0.6 vs 4.0 ± 0.9 cmH_2_O/ml *p* < 0.001) and airway driving pressure (9.2 ± 2.1 vs 15.1 ± 3.2 cmH_2_O, *p* < 0.0001) had increased significantly in all groups equally, confirming the development of mild ALI (Figure 1). The experimental setup in respect to V_T_ and V_E_ was achieved (Table 3). There was a significant drop in MAP after induction of ALI, but hemodynamic parameters (MAP, HR and CI) remained stable thereafter and were similar in all groups (Table 2). Respiratory variables (V_T_, V_E_, and RR) were similar in all groups at baseline and at the initiation of ALI.

**TABLE 2:** Hemodynamic measurements at baseline, ALI and after 4 h of ventilation in each group, including mean arterial pressure (MAP), heart rate (beats per minute, BPM), and cardiac index. Values are expressed as mean ± SD. Baseline data given for reference only, but not included into statistical analysis. There were no significant differences between groups. * denotes significance between ALI and 4 h measurements with no significant interaction between groups.

	Group	Baseline	ALI	4 hour	2-way ANOVA
MAP (mmHg)	Conventional HV_E_ Normocap	156 ± 13	117 ± 20	120 ± 28	Time *p* = 0.51 Group *p* = 0.38
	Conventional Normocapnia	158 ± 13	129 ± 19	116 ± 29	
	Protective Normocapnia	152 ± 13	123 ± 16	134 ± 40	
	Conventional iCO_2_ Hypercapnia	150 ± 13	119 ± 20	121 ± 37	
	Protective iCO_2_ Hypercapnia	146 ± 13	129 ± 14	140 ± 23	
	Protective EDG Hypercapnia	157 ± 11	119 ± 14	126 ± 28	
Heart Rate (BPM)	Conventional HV_E_ Normocap	401 ± 44	333 ± 39	406 ± 52	Time* *p* < 0.001 Group *p* = 0.212
	Conventional Normocapnia	422 ± 36	373 ± 56	428 ± 64	
	Protective Normocapnia	418 ± 44	376 ± 68	445 ± 63	
	Conventional iCO_2_ Hypercapnia	414 ± 43	343 ± 50	411 ± 59	
	Protective iCO_2_ Hypercapnia	404 ± 41	375 ± 55	420 ± 53	
	Protective EDG Hypercapnia	421 ± 30	365 ± 28	432 ± 55	
Cardiac Index (Lmin^−1^ m^−2^)	Conventional HV_E_ Normocap	2.50 ± 0.55	2.40 ± 0.42	2.22 ± 0.40	Time* *p* = 0.046 Group *p* = 0.96
	Conventional Normocapnia	2.55 ± 0.89	2.36 ± 0.75	2.27 ± 0.71	
	Protective Normocapnia	2.59 ± 0.64	2.51 ± 0.56	2.19 ± 0.44	
	Conventional iCO_2_ Hypercapnia	2.46 ± 0.48	2.44 ± 0.67	2.31 ± 0.70	
	Protective iCO_2_ Hypercapnia	2.47 ± 0.67	2.45 ± 0.59	2.08 ± 0.38	
	Protective EDG Hypercapnia	2.33 ± 0.41	2.75 ± 1.10	2.24 ± 0.85	

**TABLE 3:** Respiratory measurements at baseline, ALI and 4 h of ventilation in each group, including tidal volume (V_T_, ml/kg), respiratory rate (RR, breaths per minute, min^−1^), minute ventilation (V_E_, ml/min), respiratory system elastance (cmH_2_O/L) and transpulmonary driving pressure (cmH_2_O). Values are expressed as mean ± SD. Baseline data given for reference only, but not included into statistical analysis. * denotes significant difference vs ALI measurements; numbers denote significant differences between groups.

	Group	Baseline	ALI	4 hours	2-way ANOVA
V_T_ (ml/kg)	1 Conventional HV_E_ Normocap	7.9 ± 0.7	8.6 ± 1.0	12.3 ± 1.4*^3,5,6^	Time *p* < 0.001 Group *p* < 0.001
	2 Conventional Normocapnia	8.3 ± 0.7	8.2 ± 0.6	13.4 ± 1.2*^3,5,6^	
	3 Protective Normocapnia	8.3 ± 0.8	8.1 ± 0.7	8.4 ± 1.4^1,2^	
	4 Conventional iCO_2_ Hypercap	8.4 ± 0.9	8.5 ± 0.6	10.9 ± 1.7*^5,6^	
	5 Protective iCO_2_ Hypercapnia	8.3 ± 0.5	8.9 ± 1.4	7.8 ± 0.7^1,2,4^	
	6 Protective EDG Hypercapnia	8.3 ± 0.6	8.3 ± 0.7	8.1 ± 1.3^1,2,4^	
Interaction Time × Group *p* < 0.001					
RR (min^−1^)	1 Conventional HV_E_ Normocap	72 ± 19	72 ± 18	72 ± 16^2,4^	Time *p* < 0.001 Group *p* = 0.004
	2 Conventional Normocapnia	69 ± 14	69 ± 16	42 ± 10 *^1,3,5^	
	3 Protective Normocapnia	68 ± 13	68 ± 13	92 ± 14 *^2,4,5,6^	
	4 Conventional iCO_2_ Hypercap	68 ± 16	69 ± 18	42 ± 11 *^1,2,3,5^	
	5 Protective iCO_2_ Hypercapnia	68 ± 13	68 ± 13	68 ± 19^2,3,4^	
	6 Protective EDG Hypercapnia	68 ± 11	68 ± 13	52 ± 26^3^	
Interaction Time × Group *p* < 0.001					
V_E_ (ml/min)	1 Conventional HV_E_ Normocap	252 ± 67	275 ± 75	367 ± 104*^2,4,5,6^	Time *p* = 0.350 Group *p* = 0.018
	2 Conventional Normocapnia	261 ± 61	260 ± 76	254 ± 56^1^	
	3 Protective Normocapnia	256 ± 65	252 ± 64	347 ± 91*^4,6^	
	4 Conventional iCO_2_ Hypercap	258 ± 69	267 ± 76	204 ± 68*^3,1^	
	5 Protective iCO_2_ Hypercapnia	261 ± 58	279 ± 73	245 ± 66^1^	
	6 Protective EDG Hypercapnia	253 ± 36	256 ± 57	191 ± 107^3,1^	
Interaction Time × Group *p* < 0.001					
Respiratory System Elastance (cmH_2_O/ml)	1 Conventional HV_E_ Normocap	2.6 ± 0.5	3.8 ± 0.8	4.9 ± 2.3	Time *p* = 0.315 Group *p* = 0.471
	2 Conventional Normocapnia	2.4 ± 0.4	3.8 ± 0.7	3.6 ± 1.0	
	3 Protective Normocapnia	2.5 ± 0.7	4.2 ± 0.7	4.5 ± 0.8	
	4 Conventional iCO_2_ Hypercap	2.6 ± 0.7	4.3 ± 1.2	3.9 ± 1.4	
	5 Protective iCO_2_ Hypercapnia	2.3 ± 0.5	3.7 ± 0.7	4.2 ± 0.8	
	6 Protective EDG Hypercapnia	2.4 ± 0.4	4.1 ± 1.1	4.8 ± 1.8	
Interaction Time × Group *p* = 0.263					
Trans-pulmonary Driving pressure (cmH_2_O)	1 Conventional HV_E_ Normocap	7.9 ± 2.1	13.1 ± 3.6	23.0 ± 7.6*^3,4,5,6^	Time *p* < 0.001 Group *p* = 0.078
	2 Conventional Normocapnia	7.0 ± 1.6	12.6 ± 3.2	18.8 ± 5.1*	
	3 Protective Normocapnia	8.0 ± 3.1	13.8 ± 2.9	14.7 ± 2.9^1^	
	4 Conventional iCO_2_ Hypercap	7.3 ± 1.7	14.5 ± 4.0	16.7 ± 5.3^1^	
	5 Protective iCO_2_ Hypercapnia	6.9 ± 2.3	13.2 ± 2.8	12.8 ± 3.4^1^	
	6 Protective EDG Hypercapnia	7.9 ± 2.3	14.3 ± 3.6	16.1 ± 6.0^1^	
Interaction Time × Group *p* = 0.001					

### Gas Exchange

**FIGURE 2:**
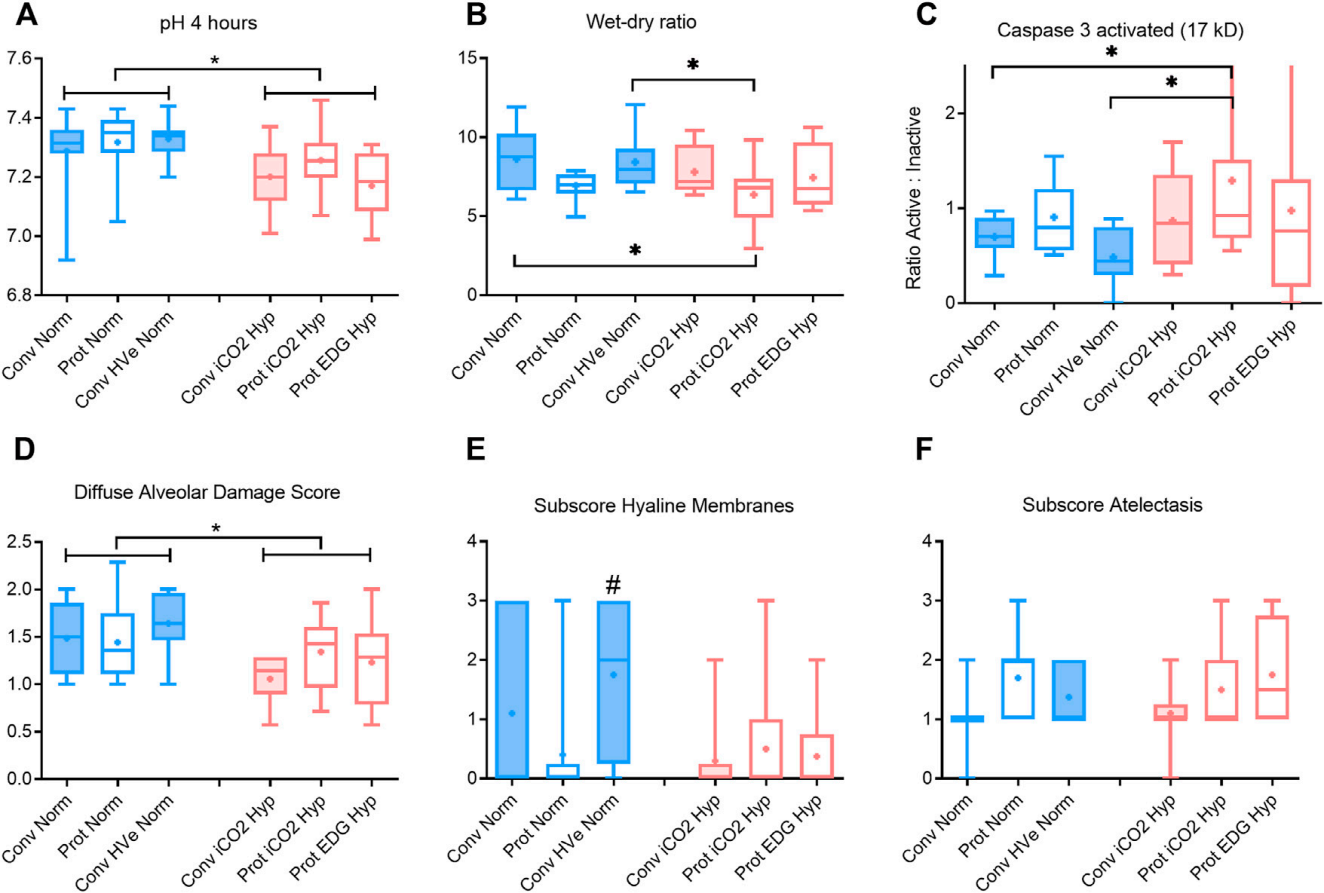
pH, wet-dry ratio, caspase activation and alveolar damage scoring after 4 h of ventilation boxplot of IQR, mean, median, min-max (whiskers). Closed boxes: high tidal volumes; open boxes: low tidal volumes; blue color: normocapnia; red color: hypercapnia. * denotes significant differences between groups # denotes significant difference to all other groups.

Gas exchange parameters were similar at baseline and at establishment of ALI in all groups with P/F ratios that would be compatible with mild ARDS in patients. The PaCO_2_ targets were achieved in all groups and by design, the hypercapnia groups had higher PaCO_2_ than the normocapnia groups. The hypercapnia group had somewhat greater acidemia than the normocapnia groups) (Figure 2A).with the lowest pH in the Protective Endogenous Hypercapnia group (*p* < 0.001) (Table 4).

#### Comparison of Conventional and Protective Ventilation Targeting Normocapnia

There were no differences in oxygenation among groups (Table 4). The V_T_ and delta_TP_ (transpulmonary driving pressure) were lower in protective vs conventional with normal or highV_E_ injurious ventilation (*p* < 0.001) (Table 3). Protective Normocapnia trended to a reduced W/D ratio compared to Conventional Normocapnia and Conventional HighV_E_ Normocapnia that failed significance in multiple comparisons (Figure 2B). Protective Normocapnia also exhibited a lower DAD score compared to Conventional HighV_E_ Normocapnia (*p* = 0.0220), with a notable decrease in the hyaline membrane subscore (*p* = 0.0271) (Figures 2D,E). The caspase-3 activation in the lung was higher in Protective Normocapnia compared to Conventional HighV_E_ Normocapnia in lung homogenates (*p* = 0.0183), however, liver or kidney histologic injury scores were not different (data not shown). In general, reduced V_T_ in protective ventilation was more likely to result in loss of airspace (Figures 2F, 5).

#### Comparison of Protective Ventilation Targeting Normocapnia With Permissive Hypercapnia

The V_T_ and delta_TP_ were similar, but V_E_ was significantly increased with protective ventilation to achieve normocapnia. In Protective Endogenous Hypercapnia, the DAD score and W/D ratio were similar to Protective Normocapnia (Figures 2B,D), and gas exchange was not improved, as shown by equal P/F ratios after 4 h of ventilation (Figure 1F). Protective Endogenous Hypercapnia also did not alter caspase-3 expression in lung homogenates (Figure 2C) and did not improve liver or kidney histologic injury (data not shown). However, Protective Endogenous Hypercapnia produced profound hypercapnic acidosis compared to Protective Normocapnia (*p* = 0.0149) (Figure 2A), and also increased IL-10 concentrations in BALF (*p* = 0.0244) (Figure 4D).

#### Comparison of Conventional Ventilation Targeting Normocapnia With Hypercapnia

**FIGURE 3:**
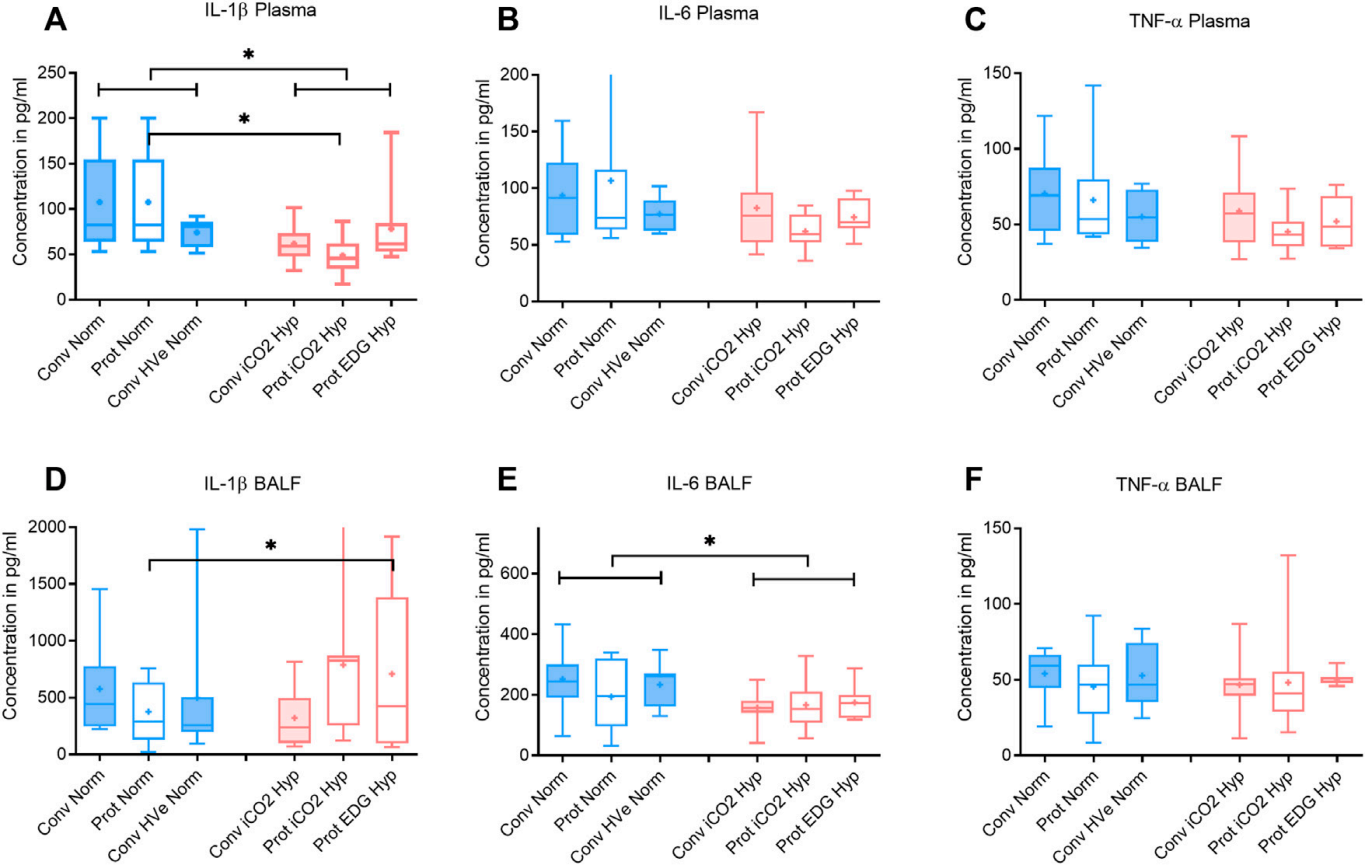
Plasma and broncho-alveolar lavage fluid (BALF) cytokines after 4 h of ventilation. Boxplot of IQR, mean, median, min-max (whiskers). Closed boxes: high tidal volumes; open boxes: low tidal volumes; blue color: normocapnia; red color: hypercapnia. * denotes significant differences between groups.

**FIGURE 4:**
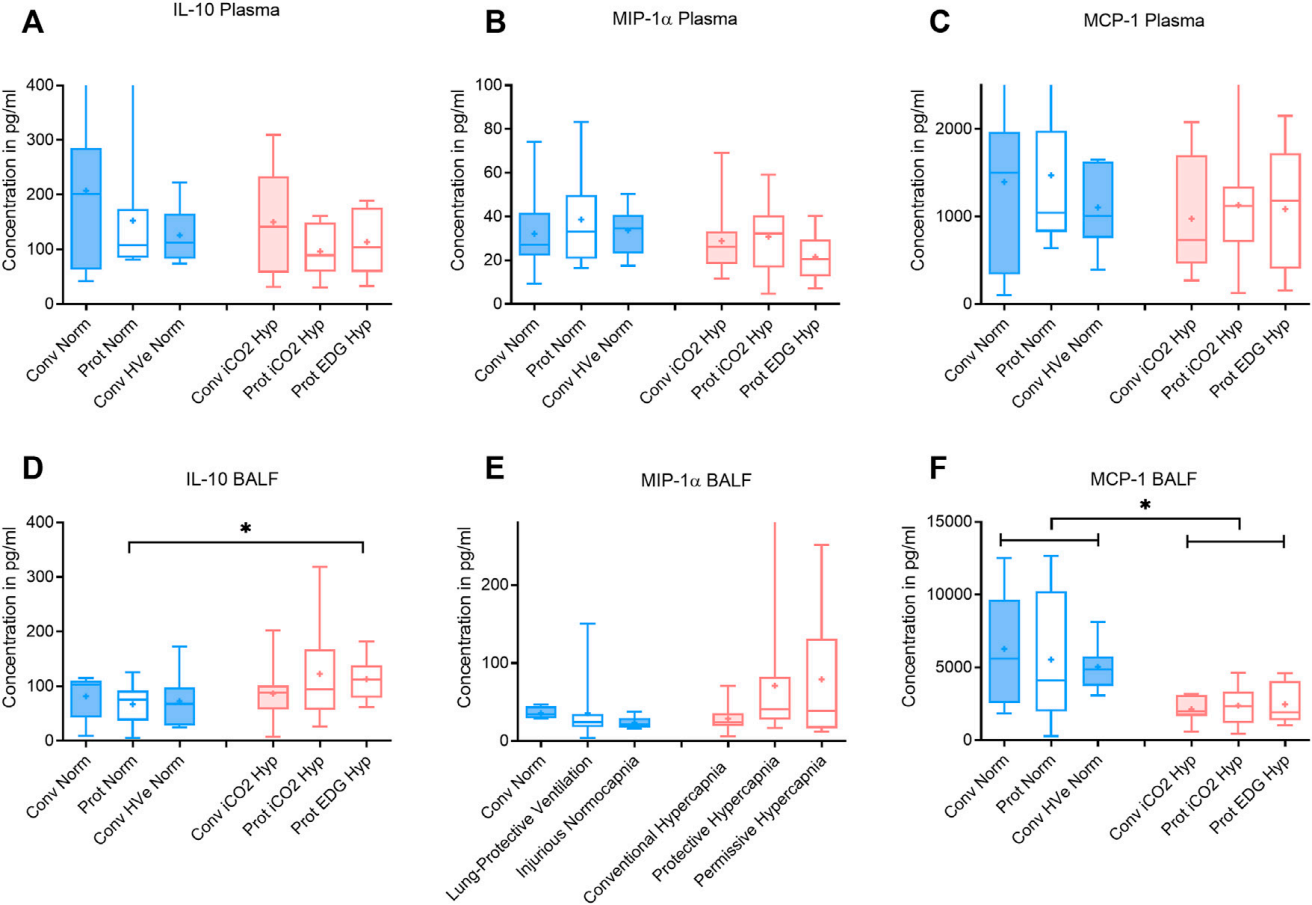
Plasma and broncho-alveolar lavage fluid (BALF) cytokines after 4 h of ventilation. Boxplot of IQR, mean, median, min-max (whiskers). Closed boxes: high tidal volumes; open boxes: low tidal volumes; blue color: normocapnia; red color: hypercapnia. * denotes significant differences between groups.

After 4 h of ventilation there were no significant differences in V_T_ or delta_TP_, although delta_TP_ increased further from the ALI time point in Conventional Normocapnia (*p* < 0.001) (Figure 1A). Adding inhaled CO_2_ in Conventional iCO_2_ Hypercapnia significantly reduced the DAD score (*p* = 0.0097) (Figure 2D), however it did not reduce the W/D ratio and did not improve gas exchange (Table 4; Figure 2B). No differences were found in caspase-3 expression (Figure 2C) or liver and kidney histologic injury (data not shown). However, there was a notable decrease in the plasma IL-1β concentrations associated with hypercapnia compared to normocapnia (*p* = 0.0015) (Figure 3A) and pulmonary IL-6 and MCP-1 concentrations were significantly reduced in the BALF (*p* = 0.0202 and *p* = 0.0030, respectively) (Figures 3E, 4F).

**TABLE 4:** Gas exchange measurements at baseline, 1 h and 4 h of ventilation in each group, including pH, partial pressure of O_2_ (PaO_2_, mmHg), and partial pressure of carbon dioxide (PaCO_2_, mmHg). Values are expressed as mean ± SD. Baseline data given for reference only, but not included into statistical analysis. * denotes significant difference vs ALI measurements; numbers denote significant differences between groups.

	Group	Baseline	ALI	4 hours	2-way ANOVA
pH	1 Conventional HVE Normocap	7.37 ± 0.12	7.37 ± 0.13	7.33 ± 0.07	Time* *p* = 0.001 Group *p* = 0.04
	2 Conventional Normocapnia	7.40 ± 0.06	7.35 ± 0.11	7.29 ± 0.14	
	3 Protective Normocapnia	7.38 ± 0.10	7.29 ± 0.05	7.32 ± 0.12	
	4 Conventional iCO2 Hypercap	7.38 ± 0.08	7.33 ± 0.10	7.20 ± 0.11*	
	5 Protective iCO2 Hypercapnia	7.38 ± 0.09	7.36 ± 0.11	7.26 ± 0.11*	
	6 Protective EDG Hypercapnia	7.37 ± 0.07	7.29 ± 0.08	7.17 ± 0.11	
Interaction Time × Group *p* = 0.207					
PaO_2_ (mmHg)	1 Conventional HVE Normocap	431 ± 92	365 ± 72	242 ± 135	Time* *p* = 0.007 Group *p* = 0.527
	2 Conventional Normocapnia	444 ± 60	315 ± 71	222 ± 134	
	3 Protective Normocapnia	420 ± 61	247 ± 89	279 ± 111	
	4 Conventional iCO2 Hypercap	442 ± 84	304 ± 110	242 ± 135	
	5 Protective iCO2 Hypercapnia	414 ± 81	304 ± 120	265 ± 94	
	6 Protective EDG Hypercapnia	421 ± 78	239 ± 68	204 ± 127	
Interaction Time × Group *p* = 0.248					
PaCO_2_ (mmHg)	1 Conventional HVE Normocap	52 ± 19	52 ± 16	55 ± 7 ^6^	Time* *p* = 0.019 Group *p* = 0.088
	2 Conventional Normocapnia	51 ± 11	53 ± 12	52 ± 9 ^6^	
	3 Protective Normocapnia	52 ± 17	61 ± 12	48 ± 9 ^4,5,6^	
	4 Conventional iCO2 Hypercap	50 ± 14	53 ± 19	68 ± 8 *^3^	
	5 Protective iCO2 Hypercapnia	50 ± 8	52 ± 13	68 ± 15*^3^	
	6 Protective EDG Hypercapnia	50 ± 11	57 ± 11	71 ± 14*^3^	
Interaction Time × Group *p* = 0.002					

#### Comparison of Protective Ventilation Targeting Normocapnia With Hypercapnia

Hypercapnia mediated by inhaled CO_2_ did not reduce the DAD score or the W/D ratio, and did not improve gas exchange compared to normocapnia if lung-protective settings were applied (Table 4; Figures 2B,D). The Protective iCO_2_ Hypercapnia group also did not differ in caspase-3 expression in lung homogenates (Figure 2C) or liver or kidney histologic injury (data not shown). However, plasma IL-1β concentrations were markedly reduced in Protective iCO_2_ Hypercapnia compared to Protective Normocapnia (*p* = 0.0042) (Figure 3A).

#### Comparison of Protective Ventilation Targeting Hypercapnia Induced via Inhaled CO_2_ With Permissive Endogenous Rise

The V_T_ and delta_TP_ were similar between groups, but V_E_ and pH were reduced in Protective Endogenous Hypercapnia (Table 4; Figures 1A, 2A). No differences were found in DAD score, W/D ratio, oxygenation or caspase-3 expression between protectively ventilated animals with endogenously or exogenously induced hypercapnia (Table 4; Figure 2). Protective iCO_2_ Hypercapnia also did not alter the inflammatory cytokine profile in plasma and BALF (Figures 3, 4). While Protective Endogenous Hypercapnia did not improve kidney histologic injury, it reduced liver histologic injury (0; 0–0.25) compared to Protective iCO_2_ Hypercapnia (1.5; 0–2) (*p* = 0.035).

## Discussion

The purpose of this study was to differentiate whether the main mechanism by which VALI is attenuated in lung-protective ventilation is attributable to the limitation of mechanical driving forces to the lung itself, or the increase in PaCO_2_ as a consequence of reduced minute ventilation. While gas exchange was impaired equally in all groups at 4 h of ventilation after establishing experimental ALI, we demonstrated that lung-protective ventilation targeting normocapnia limited histologic injury and the W/D lung ratio, and also increased caspase-3 activation compared to conventional ventilation, and especially injurious ventilation with high V_T_ and V_E_. Again this proves important evidence that relevant injury to the lungs happens before it can be detected by clinical means, i.e., changes in gas exchange or respiratory mechanics. During ventilation with high V_T_ and high delta_TP_, hypercapnia decreased histologic injury and pro-inflammatory cytokines in the plasma and BALF compared to normocapnic conditions. Hypercapnia during lung-protective ventilation had only small benefits in preventing cytokine activation as compared to normocapnia, regardless whether it was induced by inhaled CO_2_ or endogenous rise during permissive hypoventilation. However, respiratory acidosis in Protective Endogenous Hypercapnia caused an increase in BALF IL-10 concentrations, possibly exerting protective effects in distant organs.

### Does Lung-Protective Ventilation With Low Tidal Volumes Cause Less VALI Than Conventional ventilation in the State of Normocapnia?

This question may seem odd in the face of numerous experimental and clinical studies that have proven the superiority of a ventilatory concept in which stress and strain are reduced by limiting distending volumes and pressures to the lung. However, in most investigations, hypercapnia has been regarded as an undesirable side effect. Strategies have even been developed for extracorporeal CO_2_ removal (ECCO2R), with quite dissimilar outcomes and no general recommendation for its use in current guidelines (Combes et al., 2017).

When compared to high V_T_ ventilation, lung-protective low V_T_ settings reduced histologic injury and the formation of hyaline membranes, which are fibrous eosinophilic structures made of fibrin, collagen, elastin and cellular debris from mechanical strain on the lungs (Castro, 2006). The formation of hyaline membranes along alveolar walls disrupts gas exchange by creating an additional diffusional barrier through which gas exchange must occur, thereby potentially worsening oxygenation (Henzler et al., 2011). Importantly, high V_T_ is associated with increased transpulmonary driving pressures and mechanical power delivered to the lung, which have been shown to worsen VALI in experimental (Santos et al., 2018) and clinical studies (Amato et al., 2015). Our results are consistent in that delta_TP_ was higher in the high V_T_ groups exhibiting lung damage. We had previously demonstrated that transpulmonary pressure and not respiratory effort is the main determinant of VALI (Henzler et al., 2019); these findings were confirmed, since the higher RR and V_E_ in conventional ventilation (Conventional HighV_E_ Normocapnia group) did not further increase lung injury.

Protective Normocapnia also significantly increased caspase-3 activation compared to Conventional HighV_E_ Normocapnia (Figure 1). Caspase-3 is a pro-apoptotic protein representing the final step in the apoptosis common pathway onto which both the intrinsic and extrinsic apoptotic pathways converge. Lung-protective ventilation has previously been shown to reduce apoptosis in the lungs by TUNEL staining (Syrkina et al., 2008), which represents the detection of total caspase regardless of activity. In contrast, we determined the ratio of uncleaved (inactive) to cleaved (active) form by Western blotting. The reason for higher caspase-3 activation during Protective Normocapnia may be that apoptosis represents a protective rather than a harmful mechanism suggesting that it is more protective to induce programmed cell death by apoptosis than traumatic cell death by necrosis. It seems quite possible that the enhanced apoptotic activity is a product of limiting mechanical stretch without at the same time inhibiting a biochemical mechanism in the cells. This is supported by the observation that cytokine concentrations in the plasma and BALF of all animals were similar in the three normocapnia groups. Taken together, these findings indicate that VALI may be attenuated by limiting mechanisms of mechanical trauma in the lung that cause increased stretch and strain.

### Does Lung-Protective Ventilation With Permissive Hypercapnia Cause Less VALI Than Lung-Protective Ventilation Targeting Normocapnia?

Protective ventilation with Low V_T_ and low delta_TP_ allowing less V_E_ (Protective Endogenous Hypercapnia) did not further reduce VALI compared to higher RR and V_E_ (Protective Normocapnia). Permissive, low V_E_ associated increase in PaCO_2_ did not provide any further decrease in lung weight, histologic lung injury or improvement in gas exchange, and did not alter caspase-3 activation (Figure 2C). This head-to-head comparison between inhaled CO_2_ and permissive, endogenous hypercapnia has not been investigated before and confirms previous investigations that limiting delta_TP_ is more important in protecting the lung than the injurious impact of a ventilatory pattern induced when there is a high work of breathing (Henzler et al., 2019). Vaporidi et al. (2008) had investigated the effect of V_T_ and RR in mice and found that reducing RR was more effective in prevention of VILI than reducing V_T_ in ventilated mice with equal PaCO_2_ target. However, these were previously healthy animals without ALI and transpulmonary pressures were not monitored. Recently, a meta-analysis of patient-level data from observational and randomized studies investigated the influence of RR, driving pressure (delta_P_) and mechanical power on mortality in >4.500 pts. with ARDS (Costa et al., 2021). They found that there is a trade-off between reducing V_T_ and increasing RR to facilitate protective ventilation. RR was an independent predictor of death (odds ratio 1.15 (10.6, 1.25)); delta_P_ (odds ratio 1.31 (1.14, 1.5)) and mechanical power (odds ratio 1.24 (1.15, 1.33)) were even greater predictors of mortality. Using isocapnic curves, reducing RR (and therefore increasing delta_P_) reduced the odds ratio of death in patients with normal respiratory system compliance, but increased the odds ratio of death in patients with low compliance (Costa et al., 2021) if V_T_ < 7 ml/kg PBW were applied. In this respect, our results are not conflicting and may serve as the physiological basis to explain their results.

It is worth noting that the Protective Endogenous Hypercapnia group had the greatest degree of hypercapnic acidosis. This decrease in pH was associated with an increase in pulmonary IL-10 concentrations in BALF (Figure 4D). IL-10 is a potent anti-inflammatory mediator that is released in response to pro-inflammatory mediators that are produced secondary to tissue trauma (Goodman et al., 1996).

### Does Exogenous CO_2_ Attenuate VALI During Conventional Ventilation Using High Tidalvolumes?

Compared to Conventional Normocapnia, adding inhaled CO_2_ significantly reduced histologic lung injury, but did not reduce pulmonary edema, improve gas exchange, or enhance caspase-3 activation (Figure 2). Since both the Conventional Normocapnia and Conventional iCO_2_ Hypercapnia groups were ventilated with a high V_T_ and delta_TP_, a reduction in pulmonary edema and altered caspase-3 activity were not expected, which was in keeping with our findings from the first research question and the model of lung injury used in this study.

However, exogenous CO_2_ in the Conventional iCO_2_ Hypercapnia group significantly reduced pro-inflammatory cytokines in the systemic circulation (IL-1β) and in the pulmonary lavage fluid (IL-6 and MCP-1) (Figures 3, 4). MCP-1 is a potent chemoattractant substance that recruits pro-inflammatory cells to the site of injury and can be leased by epithelial cells, monocytes, and endothelial cells to signal tissue injury (Deshmane et al., 2009). The decreased MCP-1 suggests that hypercapnia has the potential to reduce recruitment of monocytes and other immune cells (T-cells, macrophages and dendritic cells) to the injured lung. The reduction in pulmonary MCP-1 may be related to the decreased pulmonary IL-6, given that many of the recruited immune cells release an abundance of IL-6 to potentiate the local inflammatory response. Peltekova et al. (2010) also demonstrated a similar decrease in MCP-1 and IL-6 in a mouse model of lung injury treated with hypercapnia (Peltekova et al., 2010). A recent investigation in previously healthy pigs with unilateral ligation of the pulmonary artery (Marongiu et al., 2021) found that inhaled CO_2_ 5% prevented an inflammatory reaction otherwise developing in both lungs. Of note, the driving pressures were significantly reduced in the iCO_2_ 5% group. The alveolar and arterial CO_2_ was double in the intervention group and ventilation-perfusion relationships (Va/Q) were not measured; it remains unclear whether the observed effects were predominately caused by the exogenous CO_2_ or by a more favorable Va/Q allowing protective ventilation settings (hen-or-egg theorem) (Marongiu et al., 2021).

While cytokine “spillover” theories have previously been proposed as a gateway from respiratory failure to systemic organ failure (Plötz et al., 2004), the decreased systemic IL-1β in the Conventional iCO_2_ Hypercapnia group is unlikely related to pulmonary IL-1β since it was not different from the Conventional Normocapnia group. It suggests that the reduced IL-1β in the systemic circulation may be related to the global effects of hypercapnia on tissues beyond the lung. Together, these findings suggest that hypercapnia can be protective even in the presence of ventilation conditions with excessive stretch and strain such as those with high tidal volume. Importantly, these results also support the notion that hypercapnia is more protective at the cellular level with its anti-inflammatory effects than at the level of tissue mechanics.

### Does Exogenous CO_2_ Attenuate VALI During Lung Protective Ventilation With Low Tidal Volumes?

Adding CO_2_ to lung-protective ventilation with a low V_T_ in the Protective iCO_2_ Hypercapnia group did not exert an additional benefit compared to Protective Normocapnia. The hypercapnia did not further reduce histologic lung injury or pulmonary edema, and did not enhance caspase-3 activity or improve gas exchange (Figure 2). Given that both groups were ventilated with low V_T_ (8 ml/kg) and the forces on the lung were equally limited, the groups only differed in the PaCO_2_ target. As such, significant differences in VALI could not be expected. However, Protective iCO_2_ Hypercapnia reduced plasma IL-1β concentrations compared to Protective Normocapnia, once again confirming the anti-inflammatory benefits of hypercapnia. Coupled with our findings from research question 3, these results indicate that hypercapnia may protect the lungs from pro-inflammatory chemical mediators independent of ventilation settings. However, it remains unclear how hypercapnia can attenuate systemic cytokines but not pulmonary cytokines if the exogenous CO_2_ is applied to the lungs through ventilation. Taken together, these findings support the notion that hypercapnia likely plays a minor role in limiting mechanical damage but may play a key role at the intracellular level by attenuating the biotrauma (pro-inflammatory) component of VALI.

### Is There an Added Effect if Hypercapnia Is Caused by Exogenous CO_2_ Instead of Permissive Hypercapnia With Endogenous Rise in CO_2_?

**FIGURE 5:**
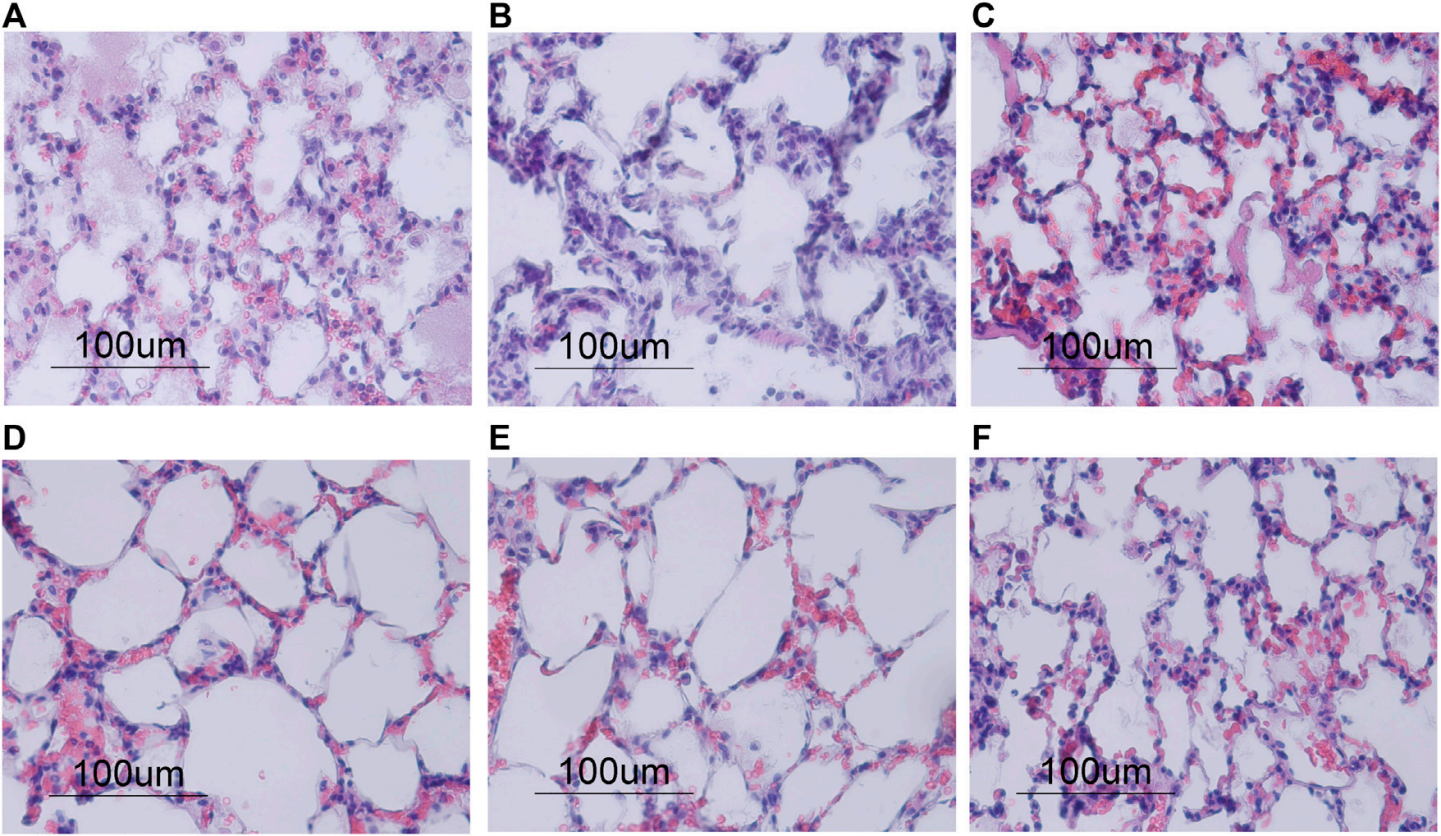
Morphological changes in lung tissue stained with hematoxylin and eosin after 4 h of ventilation, shown in ×40 magnification. **(A–C)** Normocapnia groups showed an increase in polymorphonuclear (PMN) cell infiltration compared to the hypercapnia groups **(D–F)**. Conventional Normocapnia **(A)** and Conventional HV_E_ Normocapnia **(C)** showed significant pulmonary edema (e), PMN cell infiltrates (p), hemorrhages (h), with prominent hyaline membranes (hm) in Conventional HV_E_ Normocapnia **(C)**. Protective Normocapnia **(B)** showed a reduction in air space size (a). **(D–F)** Conventional iCO_2_ Hypercapnia **(D)** and Protective iCO_2_ Hypercapnia **(E)** showed larger and well-inflated air spaces (a) and less PMN infiltrates (p), however both produced hemorrhages (h). Protective EDG Hypercapnia **(F)** produced a loss in air spaces (a), some pulmonary edema (e) and PMN infiltrates (p).

Our findings indicate that there is no added effect of hypercapnia by exogenous CO_2_ (Protective iCO_2_ Hypercapnia) compared to the hypercapnia by endogenous CO_2_ observed in the Protective Endogenous Hypercapnia group. Exogenous CO_2_ did not improve gas exchange, histologic lung injury, pulmonary edema or apoptotic activity (Figures 2–5). However, the significant reduction in liver histologic injury is a novel finding. Li et al. previously showed that exogenously induced hypercapnia improves liver histopathologic scores compared to normocapnia in a rat model of hepatic ischemia-reperfusion injury (Li et al., 2010). These findings further support the potentially important and perhaps poorly understood role of hypercapnia in attenuating histopathologic injury in the liver. Given that both groups were subject to equal PaCO_2_ levels (achieved by different methods), the only difference was the somewhat lower pH in Permissive Hypercapnia. It is speculative to conclude and cannot directly be derived from our data that acidosis has an effect greater or independently from hypercapnia (Ismaiel and Henzler, 2011).

### Limitations of the Study

While this study yielded important results that will contribute to better understanding the role of lung-protective ventilation and hypercapnia in attenuating VALI, it was not without its limitations. First, only male rats were used in this study, as this was traditionally believed to minimize the contribution of hormonal variability that can potentially result from including female rats in the study. However, it is possible that including only male rats may limit the generalizability of the results of this study. Second, the current experimental model of ALI in this study by endotracheal instillation of HCl (Imai et al., 2003; Henzler et al., 2011) simulates the aspiration of gastric contents that can often lead to ALI, though the authors acknowledge that ALI induced or aggravated by mechanical ventilation may have a higher incidence. In addition, it is possible that the short duration of ventilation (a total of 4 h, 1 h to establish acute lung injury *per definitionem* and 3 h in each respective group) may not have been long enough to identify additional benefits of the different ventilation modalities, especially in looking for an improvement in gas exchange. However, several previous investigations have shown improvements in gas exchange, inflammatory parameters and lung damage after 4 h of ventilation (Chiumello et al., 1999; Sinclair et al., 2002). In our study, a 4-h ventilation period was sufficient to detect early anti-inflammatory changes in cytokine profiles and lung damage, but not improvements in gas exchange. It’s possible that a 6-h ventilation period could have unveiled more benefits of lung-protective ventilation and hypercapnia (Laffey et al., 2004).

Another limitation of our study may be related to the markers used to quantify the protective and beneficial effects of hypercapnia and hypercapnic acidosis. We have primarily investigated the changes in the cytokine profiles in the BALF and plasma, and caspase-3 activity in lung homogenates. It is possible that performing additional experiments to quantify free radical and reactive oxygen species production via changes in xanthine oxidase activity could have offered additional information about the protective and anti-inflammatory benefits of hypercapnia and hypercapnic acidosis, which had already been demonstrated before (Shibata et al., 1998).

Finally, the findings of our study may be limited to the ventilation settings, specifically those related to the PaCO_2_ targets. It is possible that the PaCO_2_ targets for normocapnia and hypercapnia were not distinct enough, and that the normocapnia target range (40–55 mmHg) may have been too high for normocapnia and likely approaching hypercapnia. Similar PaCO_2_ targets have been used for normocapnia, although the PaCO_2_ targets for hypercapnia were almost double those used in the present study (Peltekova et al., 2010). Similarly, V_T_ appeared to be higher in Conventional Normocapnia vs Hypercapnia, although these differences were not significant. We cannot rule out the possibility that the observed differences in biological injury were influenced by differences in V_T_ as well. We chose 8 ml/kg as the target volume for our low V_T_ groups for experimental reasons, although several data suggest 6–8 ml/kg predicted body weight as a safe range of tidal volumes for most protective settings (Vaneker et al., 2007; Güldner et al., 2015). However, there is no experimental data suggesting that 8 ml/kg is more protective than 6 ml/kg and the concept of using predicted body weight instead of actual body weight is cumbersome in rodent research. Choosing V_T_ was a compromise between prevention of volutrauma and mechanical power that would have increased with even higher RR to achieve the PaCO_2_ targets. Although the differentiation between V_T_ and driving pressure as to the main protective mechanism remains controversial (Amato et al., 2015), it might have been possible to detect more significant differences between our low V_T_ and high V_T_ groups if we compared 6 ml/kg vs 10–12 ml/kg.

## Conclusion

The aim of this study was to explore five key research questions about the role of hypercapnia in the prevention of VALI. Our findings indicate that there are two distinct mechanisms attached to protective ventilation in the attenuation of VALI: the limitation of mechanical damage and the limitation of biological damage. These mechanisms interact during mechanical ventilation to maximize protection against VALI. Consistent with previous investigations we found that mechanical damage is best attenuated by limiting tidal volume and transpulmonary driving pressures, to minimize excessive stretch and strain. The combined reduction of these mechanical mechanisms is believed to minimize traumatic cell death and promote apoptosis in the lung. The therapeutic effects of hypercapnia appear to extend beyond the lungs to distant organs, where the spontaneous rise in CO_2_ attenuated histologic liver injury with permissive hypercapnia. These findings suggest that combining ventilation strategies that integrate lung-protective settings that limit tidal volumes, minute ventilation and driving pressures with permissive hypercapnia as an accepted side effect may offer the best protection against VALI. Lung-protective ventilation may limit mechanical damage to the lung, while hypercapnia attenuates VALI by limiting pro-inflammatory and biochemical mechanisms of injury. When combined, both have the potential to exert a synergistic effect for prevention of VALI and its systemic effects. Our results are encouraging and hold clinical implications for the future of research on VALI and clinical practice as the medical community makes the leap from the bench to the bedside.

## Data Availability

The datasets presented in this article are not readily available but physiological and biological data from animal study will be made available by reasonable request. Requests to access the datasets should be directed to DH, mail@d-henzler.de.
