# Supplementary material for: Lung-Protective Ventilation Attenuates Mechanical Injury While Hypercapnia Attenuates Biological Injury in a Rat Model of Ventilator-Associated Lung Injury
Source: Front Physiol. 2022 Apr 21;13:814968. doi: 10.3389/fphys.2022.814968 (PMC9068936; doi:10.3389/fphys.2022.814968)
Supplement: Supplementary file 1 [file DataSheet1.docx]

**Supplementary Methods**

*Instrumentation and Experimental Protocol*

The rats were obtained from Charles River Laboratories (Saint-Constant, QC, Canada) and were given unlimited access to Prolab Rodent Chow and water. Rats were anesthetized by intraperitoneal injection of sodium pentobarbital (55 mg/kg). A thermocouple temperature probe was inserted into the aorta through the right femoral artery to facilitate the monitoring of body temperature and the measurement of cardiac output (CO) using the thermodilution principle (Physitemp Instruments Inc., Clifton, NJ, USA).^17^ The CO was normalized to the body surface area of each rat and used to calculate the cardiac index (CI). A catheter was inserted into the left jugular vein and used for the administration of intravenous medications and fluids using a syringe pump (Braun Medical, Melsungen, Germany). A continuous intravenous infusion of 20 mcg/ml remifentanil and 25 mcg/ml pancuronium at 5 ml/hour was given during the course of ventilation. The remifentanil was given at 0.4 mcg/kg/min to provide continuous analgesia and sedation^17^, and the pancuronium was given at 0.2 mg/kg/hr to provide neuromuscular blockade to facilitate controlled ventilation.

The left carotid artery was cannulated to monitor mean arterial pressure (MAP). A tracheostomy was performed using a 14G cannula that was connected to a pneumotachometer (Series 8420B, rat and guinea pig-specific, Hans Rudolph Inc., Shawnee, KS, USA) used to deliver mechanical ventilation and measure spirometry flow and tidal volume (VT). During instrumentation, all rats received oxygen therapy using 100% O2 delivered from a neonatal ventilator (Evita XL, Draeger Medical Inc., Richmond Hill, ON, Canada). The arterial catheter was connected to a physiologic pressure transducer (ADInstruments, Colorado Springs, CO, USA). All data from the pneumotachometer, pressure transducer, electrocardiogram leads and temperature probe input into the LabChart software (LabChart 6.0, ADI Instruments) used for continuous monitoring.

Complete measurements (hemodynamics, respiratory mechanics, gas exchange) were taken at baseline, 1 hour and 4 hours of ventilation. The final (4 hour) measurements also included the collection of BALF and arterial plasma, and were followed by excision of tissue samples from the lung, liver and kidney. All bodily fluids were flash frozen in liquid nitrogen and stored at -80°C until analysis. Tissue samples were fixed in 10% unbuffered formalin and subsequently embedded in paraffin, sectioned into 5 μm sections and mounted onto glass slides. The slides were stained with hematoxylin and eosin and scored by a lung pathologist and a kidney pathologist who were blind to group assignments.

*Diffuse Alveolar Damage (DAD) Lung Injury Score*

The DAD score was based on seven criteria: interstitial edema (within lung tissues), alveolar edema, hemorrhages, polymorphonuclear (PMN) cell infiltrates, atelectasis, alveolar damage, and hyaline membranes.^18,21^ The arithmetic mean of all subscores was taken as the DAD score for each lung sample. The presence and severity of each criterion was rated from 0 to 3. A score of 0 represents ‘no damage present’, 1 represents ‘mild damage, few lesions’, 2 represents ‘moderate damage, lesions in every visual field’ and 3 represents ‘severe damage, lesions ubiquitous’.

*Liver and Kidney Injury Score*

The liver tissues were assigned a score from Grade 0 to Grade 2, where Grade 0 represents ‘unremarkable changes’, Grade 1 represents ‘mild fatty changes’, and Grade 2 represents ‘moderate fatty changes’. The kidney tissues were assigned a score from Grade 0 to Grade 4, where Grade 0 represents ‘unremarkable changes’, Grade 1 represents ‘mild changes including tubular dilatation and vacuolization’, Grade 2 represents ‘scattered single tubular epithelial cell necrosis including active lymphoid infiltration’, Grade 3 represents ‘areas of tubular necrosis’ and Grade 4 represents ‘large segments of tubular necrosis with hemorrhage’.

*Wet-to-Dry Lung Ratio*

During the tissue dissection, the middle lobe of the right lung was cut off, placed in a pre-weighed glass scintillation vial, weighed again, and placed in a warming oven at 40°C for 48 hours. After that time, the dried lobe was removed from the oven and weighed. The weight of the vial when empty, when containing the wet lung, and when containing the dry lung were all recorded and used to calculate the ratio of the wet-to-dry lung and used as a measure of pulmonary edema. The weight measurements were collected using a precision balance scale (Sartoius GmBH, Gottingen, Germany).

*Tissue Homogenization Protocol*

The right upper lobe was homogenized to probe for caspase-3 activation by western blotting. The right upper lobe was cut into smaller pieces using a razor and placed in a pre-weighed 15 ml Falcon tube. The lung was weighed again and the weight recorded. Ice-cold 1X radioimmunoprecipitation assay (RIPA) buffer (Cell Signaling Technology) was purchased through New England Biolabs (Pickering, ON, Canada). The serine protease inhibitor phenylmethanesulfonylfluoride (1 mM PMSF; Sigma Aldrich, Oakville, ON, Canada) was added to the RIPA buffer prior to use (75 μl PMSF/0.01 g of tissue). A tissue homogenizer (PRO200, Monroe, CT, USA) was used at high speed for 45-60 seconds to mechanically break the lung tissue. The homogenized tissue was placed at 4°C with gentle rocking (Rocker 25 from Labnet, Edison, NJ, USA) for 15 minutes, followed by 30 seconds of vortexing and repeated. The homogenates were then subject to 3 cycles of sonication (30 seconds/cycle) using a probe ultrasonic homogenizer (4710 series, from Cole Parmer, Montreal, QC, Canada). The homogenates were centrifuged (MIKRO 120, Hettich, Beverly, MA, USA) at 8000 rpm for 10 minutes at 4°C, and the supernatant transferred into fresh tubes. This supernatant was once again vortexed, centrifuged at 13000 rpm for 20 minutes at 4°C, and the resulting supernatant was collected and stored at -20°C for future protein measurement and western blotting.

*Bradford Protein Assay*

A protein assay was performed to determine the protein concentration in the rat lung homogenates.^32^ A protein standard curve was created using bovine serum albumin (BSA; 2 mg/ml from BIO-RAD, Mississauga, ON, Canada) with five BSA standards (0, 2, 5, 10 and 15 μg of BSA), run in duplicates. Bradford Reagent Dye (500 μl), double-distilled water, 1X RIPA buffer were added to each standard for a final measuring volume of 1 ml. The lung homogenate test samples were prepared similarly in duplicates, adding 10 μl of protein, double-distilled water, and Bradford Reagent Dye (500 μl) for a final volume of 1 ml. The BSA standards and lung homogenate samples were measured on a 2802 UV/VIS spectrophotometer (UNICO, Dayton, NJ, USA) at 595 nm, and the optical density (OD) values were recorded using the UV/VIS Analyst software. Based on the measured ODs of the standards, a curve was constructed and a linear regression was used to fit the ODs and calculate the R2 value. An R2 value between 0.950 and 1.00 was considered acceptable for estimating the protein concentrations in the lung homogenates based on the measured ODs. For this study, all standard curves constructed had an R2 value between 0.960-0.990. The lung protein concentration was calculated, and the loading volume was determined based on loading 50 mcg of protein in each well during the western blot.

*Western Blotting for Caspase-3 and Actin*

To examine whether apoptotic activity was modified in the rat lung as biomarker of VALI, western blotting was used to probe for caspase-3 activation. This was determined by the expression levels of uncleaved (inactive) and cleaved (active) forms of caspase-3 in the lung homogenates. The lung homogenate samples were prepared based on the determined volumes from the protein assay (50 mcg loaded protein) and 5X sample buffer.

Prior to loading, the samples were heated for 5 minutes at 85°C to denature the proteins, and the boiled samples were loaded into a 12% SDS-PAGE resolving gel. Alongside the samples, a Precision Plus Kaleidoscope 10-band protein standard (10-250 kD, from BIORAD,

Mississauga, ON, Canada) was used. The gel gasket chamber was filled with 1X running buffer and connected to a PowerPac HC from BIO-RAD, and run at 90V and 300W for approximately 70 minutes.

When the proteins separated on the gel, the proteins were transferred onto a pure nitrocellulose membrane (Pall Corporation, Pensacola, FL, USA) in the gasket chamber (filled with 1X transfer buffer) for 2 hours at 45V. The membrane was saturated with a 5% milk solution (made with 1X TBS and 0.2% Tween; 1X TTBS) for 1 hour with gentle rocking at room temperature (Stovall Lifescience Inc., Greensboro, NC, USA).

The primary polyclonal Caspase-3 antibody (raised in rabbit) detects the uncleaved (inactive) 35 kD protein band, as well as 17 and 12 kD cleaved (active) fragments. The antibody has species cross-reactivity for human, mouse, rat and monkey (Cell Signaling Technology). The primary antibody was added at 1:1000 dilution prepared with 5% milk and 1X TTBS and incubated overnight at 4°C with gentle rocking. The primary antibody was removed, and the membrane washed with 1X TBS and TTBS. The secondary antibody (Anti-rabbit IgG, HRP-linked, Cell Signaling Technology) was added at a 1:1000 dilution prepared with 5% milk and 1X TTBS and incubated for 2 hours with gentle rocking at room temperature. The secondary antibody was removed, and the membrane was washed with 1X TBS and TTBS. The membrane was imaged using the ECL Plus western blot detection system (GE Healthcare, purchased from Fisher Scientific, Ottawa, ON, Canada). The membrane was exposed to film for 10 minutes and subsequently developed.

The primary and secondary antibodies were stripped off the membrane using Restore western blot stripping buffer from Thermo Scientific (from Fisher Scientific, Ottawa, ON, Canada). The membrane was saturated with 5% milk solution and re-probed with an actin antibody as a loading control. The membrane was incubated in a 1:400 dilution of mouse monoclonal antibody of Actin HRP-linked IgG (Santa Cruz Biotechnology Inc., Santa Cruz, CA, USA) overnight at 4°C with gentle rocking. The antibody dilution was prepared with 1X TBS and 0.2% BSA. The actin antibody was removed, and the membrane was washed with 1X TBS and TTBS and imaged using the ECL Plus detection kit. The membrane was exposed to film for 1 minute and subsequently developed.

*Densitometric Analysis of Caspase-3 Expression*

The developed films were scanned and analyzed using the Image J software (National Institues of Health, Bethesda, USA). The images were inverted and the density of the uncleaved (inactive) caspase-3 band (35 kD), cleaved (active) caspase-3 band (17 kD), and background were measured and recorded. The difference between the measured bands and the background was calculated, and the ratio of active: inactive caspase-3 was determined and used as a measure of caspase-3 activation.

*Cytokine Analysis Protocol*

The cytokines and chemokines measured were IL-1β, ICAM-1, IL-6, TNF-α, GM-CSF, IL-10, RANTES, KC, MCP-1, and MIP-1α. The BALF and arterial plasma samples collected from all animals at the end of each experiment were analyzed using the cytokine assay kit and Luminex Technology Analyzer 100 and BioPlex Manager software from BIO-RAD (Mississauga, ON, Canada). The Luminex instrument was calibrated before every experiment, and validated once every 30 days. Calibration and Validation kits (BIO-RAD) were used to test and ensure the optimal performance of the Luminex instrument. The instrument was used to run cytokine assays only when the calibration and validation were successful.

The antibody beads (containing beads for the 10 selected cytokines) were loaded into all wells and washed by vacuum filtration. Rat-specific bodily fluid buffer was added to all wells (25 μl). The standards used to calculate cytokine concentrations in the samples were prepared by reconstituting the lyophilized premixed standard powder in 500 μl of rat-specific bodily fluid buffer. A total of 8 standards were prepared starting at 10,000 pg/ml of each analyte and decreasing 4-fold with each standard. All standards and samples were loaded into a 96-well filter plate in duplicates (25 μl/well for a final volume of 50 μl/well). The standards and samples were incubated on a shaker at 500 rpm for 1 hour at room temperature, and subsequently removed from the plate by vacuum filtration.

The wells were washed with 1X wash buffer, and the detection antibody was added to the plate (25 μl/well) and incubated on a shaker at 500 rpm for 30 minutes. The antibody was removed by vacuum filtration and the plate was washed with 1X wash buffer. Streptavidin-PE was added to each well (50 μl/well) and incubated on a shaker at 500 rpm for 30 minutes. Finally, the streptavidin-PE was removed by vacuum filtration and washed with 1X wash buffer. Reading buffer (120 μl/well) was added to all wells and incubated on a shaker at 500 rpm for 5 minutes and then inserted into the Luminex instrument for reading.

A separate standard curve was produced for each cytokine measured, with a limit of detection <1 pg/ml for each cytokine. Since each standard and sample were assayed in duplicates, the mean of the measured concentrations was taken. The final dilution of each sample was 1:2, and the cytokine concentrations were calculated based on the set dilution. To ensure that an adequate standard curve was used for each analyte, the ratio of [observed/expected] x 100 standard concentrations was 100 ± 30, and all standard values were within this range.

*Statistical Analysis*

Two animals each were excluded from statistical analyses in the Permissive Hypercapnia and Injurious Normocapnia groups due to premature death. All groups had 10 rats each except for the Permissive Hypercapnia and Injurious Normocapnia groups, which had 8 rats each.
